# Premature ventricular contraction is associated with increased risk of atrial fibrillation: a nationwide population-based study

**DOI:** 10.1038/s41598-021-81229-0

**Published:** 2021-01-15

**Authors:** Yun Gi Kim, Kyung-Do Han, Jong-Il Choi, Yun Young Choi, Ha Young Choi, Jaemin Shim, Young-Hoon Kim

**Affiliations:** 1 Division of Cardiology, Korea University College of Medicine and Korea University Anam Hospital, 73 Goryeodae-ro, Seongbuk-gu, Seoul, 02841 Republic of Korea; 2 Department of Statistics and Actuarial Science, Soongsil University, Seoul, Republic of Korea

**Keywords:** Cardiovascular biology, Cardiology

## Abstract

Premature ventricular contraction (PVC) and atrial fibrillation (AF) are common arrhythmias affecting 1–2% of the general population. During PVC, retrograde ventriculo-atrial activation can occur and act like an atrial ectopy. However, the clinical significance of this phenomenon is not fully understood. We aimed to elucidate whether the clinical diagnosis of PVC can increase the risk of new-onset AF. We performed a nationwide population-based analysis using the Korean National Health Insurance Service database. A total of 9,537,713 people without prior history of PVC and AF were identified. Among these people, 4135 developed PVC in 2009, and people with and without PVC were followed until 2018. People who had PVC showed an increased risk of new-onset AF as compared with people without PVC (hazard ratio [HR] = 2.705; 95% confidence interval [CI] = 2.428–3.013; p < 0.001). The risk of ischemic stroke was also significantly increased in people with PVC (HR 1.160; 95% CI 1.048–1.284; p = 0.0041). New-onset AF developed in 72 people (19.3%) among 374 people with PVC who had ischemic stroke during their follow-up. A significant interaction was observed between PVC and age with people < 65 years at greater risk of new-onset AF for having PVC. In this observational analysis, the risk of new-onset AF and ischemic stroke was increased in people with PVC. Additional evaluation to identify AF in people with PVC can be helpful.

## Introduction

Premature ventricular contraction (PVC) is a common arrhythmia affecting 1% of the general population^1^. Early ventricular depolarization is responsible for PVC which is associated with symptoms such as palpitations, chest discomfort, sense of skipped beat, presyncope, and syncope^2,3^. Patients with PVCs can also suffer from PVC-induced cardiomyopathy or ventricular tachyarrhythmias such as ventricular tachycardia and fibrillation^2,3^. Although the clinical course of PVC is usually benign, they can cause various comorbidities in a subset of patients^4–7^.

Atrial fibrillation (AF), another common arrhythmia with an estimated prevalence of 1–2%^8,9^, is associated with impaired quality of life, presents with various symptoms such as palpitation, ischemic stroke, heart failure, and increased overall mortality^10–12^. Atrial ectopic beats originating from pulmonary veins are assumed to be an important pathophysiology of AF^13^.

The association between PVC and AF is not fully understood. In theory, retrograde ventriculo-atrial conduction can occur with PVC, which can act like atrial ectopic beats. Therefore, there is a possibility that PVC can increase the risk of AF through retrograde ventriculo-atrial conduction. Prior studies suggest the presence of PVC can increase the risk of ischemic stroke^14,15^. If PVC can increase the risk of new-onset AF, they would also increase the risk of ischemic stroke since AF is strongly associated with ischemic stroke. We aimed to evaluate whether clinical diagnosis PVC is associated with an increased risk of new-onset AF or ischemic stroke using nationwide healthcare database. Demonstration of increased risk of new-onset AF in people with PVC will also provide mechanistic insight to increased risk of stroke in people with PVC.

## Methods

### Patients

This study was based on the Korean National Health Insurance Service (K-NHIS) database to which the majority of Koreans (97.1%) are mandatory subscribers. The K-NHIS is the single medical insurer in the Republic of Korea managed by the government. Therefore, the entire population of the Republic of Korea is represented by the K-NHIS database. Various healthcare data such as baseline demographics, diagnosis codes of various diseases including AF and PVC, inpatient and outpatient service records, drug prescriptions, and mortality event are stored in the K-NHIS database. Regular nationwide health check-ups for all subscribers are provided by the K-NHIS and include (1) a health questionnaire about past medical history, alcohol consumption, smoking status, and physical activity, (2) laboratory tests, (3) and measurements of blood pressure, body weight, height, and waist circumference. Therefore, nationwide health check-ups can be used as a valuable medical research cohort with a significantly large sample size. Medical researchers with approved study protocols by the official review committee (https://nhiss.nhis.or.kr/) can use the K-NHIS database for research purpose.

In this study, people who underwent a national health check-up in 2009 were included. Data obtained from January 2002 to December 2008 were used as screening data to identify medical history such as AF, PVC, stroke, hypertension, diabetes, dyslipidemia, and heart failure. The follow-up period was from the date of the national health check-up to December 2017. Patients were excluded if they had already been diagnosed with AF, PVC, ventricular tachycardia, ventricular fibrillation, heart failure, or stroke during the screening period or if they were younger than 20 years old.

Diagnostic codes related to PVC and AF were retrieved and we were able to analyze the impact of PVC on new-onset AF. The impact of PVCs in ischemic stroke was also evaluated. The Institutional Review Board of Korea University Anam Hospital approved this specific study. The written informed consent form was waived due to the retrospective nature of the study. The study protocol strictly adheres to the ethical guidelines of the 2008 Declaration of Helsinki and legal regulations of Republic of Korea.

### Primary outcome endpoint

People who had a national health check-up in 2009 were classified into one of two groups: (1) those who were diagnosed with PVC in 2009 and (2) those who were not diagnosed with PVC in 2009. The incidence of new-onset AF and ischemic stroke was compared. The incidence of new-onset AF was defined as the number of new-onset AF cases calculated for 1000 patient * years of follow-up. The impact of the aforementioned non-genetic risk factors was evaluated in various age groups.

### Definitions

The diagnosis of PVC was based on two criteria: (1) one inpatient or outpatient record (PVC 1) or (2) two or more outpatient records or one inpatient record (PVC 2) of the International Classification of Disease, Tenth Revision (ICD-10; I49.3) codes in the K-NHIS database. People with more symptomatic PVC will likely to have subsequent outpatient clinic visit or hospital admission. Therefore, PVC diagnosed by repeat outpatient or inpatient record might represent more symptomatic form of PVC.

Diagnosis of AF required two outpatient records or one inpatient record (I48). The robustness of this definition has been validated in previous studies^16,17^. One or more outpatient or inpatient records was required for the diagnosis of ischemic stroke (I63, I64). In the current study, transient ischemic attack was not included in ischemic stroke. The exact diagnosis codes for PVC, AF, and other diseases are described in Supplementary Table S1.

Body weight status was classified into five groups according to body mass index (BMI): BMI < 18.5 kg/m^2^, 18.5 ≤ BMI < 23.0, 23.0 ≤ BMI < 25.0, 25.0 ≤ BMI < 30.0, and BMI ≥ 30.0. Smoker was defined as those who smoked at least 100 cigarettes in their lifetime and continued smoking within 1 month of the 2009 national health check-up. Ex-smokers were those who smoked at least 100 cigarettes in their lifetime but had not smoked within 1 month of the 2009 national health check-up, and never-smokers were those who smoked < 100 cigarettes in their lifetime. Based on the total amount of alcohol intake per week, patients were classified into three groups: (1) nondrinker, 0 g of alcohol per week; (2) mild- to moderate-drinker, less than 210 g but more than 0 g of alcohol per week; and (3) heavy-drinker, 210 g or more of alcohol per week. Diagnosis of diabetes was made for participants of the 2009 national health check-up who had impaired fasting sugar > 126 mg/dl or prior history of physician-diagnosed diabetes. Hypertension was defined as those who were previously diagnosed with hypertension or had either systolic blood pressure ≥ 140 mmHg or diastolic blood pressure ≥ 90 mmHg during 2009 national health check-up. Dyslipidemia was defined as a prior history of dyslipidemia diagnosis by a physician. Estimated glomerular filtration rate < 60 ml/min/1.73 m^2^ was the diagnostic criteria for chronic kidney disease.

### Statistical analysis

For continuous variables, the Student’s t-test was used; results are described as mean ± standard deviation. Categorical variables are presented as percentile values and were compared with a chi-square test or Fisher’s exact test as appropriate. Cumulative incidence curve analysis with log-rank t test and death as a competing risk was used to compare the incidence of new-onset AF and ischemic stroke in people with and without PVC. Univariate and multivariate Cox regression analyses were performed to calculate the hazard ratio (HR) and its 95% confidence interval (CI). In total, three multivariate models were used: (1) model 1: adjusted for age and sex; (2) model 2: adjusted for age, sex, smoking status, alcohol consumption status, regular physical activity, and BMI; (3) model 3: adjusted for age, sex, smoking status, alcohol consumption status, regular physical activity, BMI, hypertension, diabetes mellitus, dyslipidemia, and chronic kidney disease; (4) model 4: adjusted for age, sex, smoking status, alcohol consumption status, regular physical activity, BMI, hypertension, diabetes mellitus, dyslipidemia, chronic kidney disease, myocardial infarction, mitral stenosis, and aortic stenosis. Age and BMI was adjusted as continuous variables. Start of follow-up was January 1, 2009 for people without PVC and the time of the diagnosis of PVC for people with PVC. If the patient who did not have PVC in 2009 (and therefore included in the non-PVC group) but developed PVC after 2009, he or she was censored at the that time period. The event date of new-onset AF was the date of the second outpatient input or the first inpatient input of ICD-10 code for AF. All tests were two-tailed, and p values ≤ 0.05 were considered statistically significant. All statistical analyses were performed with SAS version 9.2 (SAS Institute, Cary, NC, USA).

## Results

### Patients

**Figure 1:**
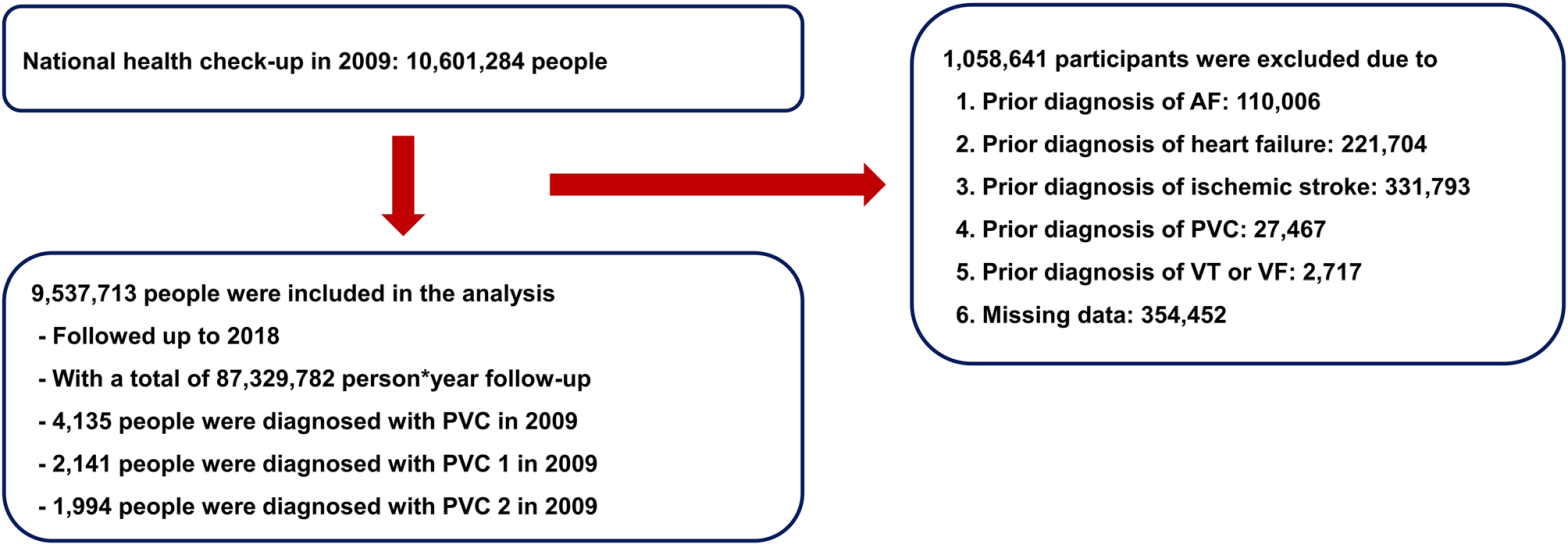
Study flow. *AF* atrial fibrillation, *PVC* premature ventricular contraction, *VF* ventricular fibrillation, *VT* ventricular tachycardia.

A total of 10,601,284 people underwent a national health check-up in 2009. People who were (1) under 20 years of age or (2) had a prior history of heart failure, PVC, AF, ischemic stroke, ventricular tachycardia, or ventricular fibrillation were excluded. In the analysis, 9,537,713 people were included. In total, 4135 people were diagnosed with PVC with 2141 and 1994 people having PVC 1 (one record) and PVC 2 (two outpatient record or one inpatient record), respectively, in 2009. The study flow is shown in Fig. 1. Baseline clinical characteristics of the cohort are summarized in Table 1. Significant difference in demographics among people with and without PVC was observed. People who developed PVC were significantly older; more likely to be female/non-drinker/non-smoker; and had a higher prevalence of hypertension, diabetes mellitus, and dyslipidemia.

**Table 1:** Baseline demographics of patients with and without PVC.

	No PVC	All PVC (PVC 1 + PVC 2)	PVC 1	PVC 2	p value
	n = 9,533,578	n = 4135	n = 2141	n = 1994	(no PVC vs. all PVC)
Male sex	5,306,708 (55.66%)	1966 (47.55%)	1025 (47.87%)	941 (47.19%)	< 0.001
Age ≥ 65 years	1,027,296 (10.78%)	872 (21.09%)	403 (18.82%)	469 (23.52%)	< 0.001
**Alcohol consumption**					< 0.001
Non-drinker	4,781,029 (50.15%)	2582 (62.44%)	1315 (61.42%)	1267 (63.54%)	
Mild- to moderate-drinker	3,973,810 (41.68%)	1303 (31.51%)	704 (32.88%)	599 (30.04%)	
Heavy-drinker	778,739 (8.17%)	250 (6.05%)	122 (5.70%)	128 (6.42%)	
**Smoking status**					< 0.001
Non-smoker	5,592,120 (58.66%)	2716 (65.68%)	1406 (65.67%)	1310 (65.70%)	
Ex-smoker	1,359,113 (14.26%)	742 (17.94%)	379 (17.70%)	363 (18.20%)	
Current-smoker	2,582,345 (27.09%)	677 (16.37%)	356 (16.63%)	321 (16.10%)	
Regular physical activity	1,721,006 (18.05%)	893 (21.60%)	464 (21.67%)	429 (21.51%)	< 0.001
Hypertension	2,326,074 (24.40%)	1946 (47.06%)	791 (36.95%)	1155 (57.92%)	< 0.001
Diabetes mellitus	752,535 (7.89%)	424 (10.25%)	185 (8.64%)	239 (11.99%)	< 0.001
Dyslipidemia	1,609,858 (16.89%)	1111 (26.87%)	471 (22%)	640 (32.10%)	< 0.001
Myocardial infarction	29,554 (0.31)	94 (2.27)	40 (1.89)	53 (2.68)	< 0.001
Mitral stenosis	1907 (0.02)	7 (0.17)	3 (0.14)	5 (0.25)	< 0.001
Aortic stenosis	2860 (0.03)	15 (0.36)	9 (0.41)	6 (0.3)	< 0.001
**BMI**					< 0.001
BMI < 0 18.5	359,286 (3.77%)	116 (2.81%)	59 (2.76%)	57 (2.86%)	
18.5 ≤ BMI < 0 23	3,767,938 (39.52%)	1516 (36.66%)	820 (38.30%)	696 (34.90%)	
23 ≤ BMI < 0 25	2,343,862 (24.59%)	1067 (25.80%)	556 (25.97%)	511 (25.63%)	
25 ≤ BMI < 0 30	2,732,422 (28.66%)	1294 (31.29%)	638 (29.8%)	656 (32.90%)	
30 ≤ BMI	330,070 (3.46%)	142 (3.43%)	68 (3.18%)	74 (3.71%)	
Age	46.07 ± 13.65	52.71 ± 13.31	51.2 ± 13.57	54.34 ± 12.83	< 0.001
Height	164.24 ± 9.17	162.68 ± 9.05	163.07 ± 9.13	162.25 ± 8.96	< 0.001
Weight	64.09 ± 11.69	63.46 ± 11.12	63.45 ± 11.21	63.46 ± 11.02	0.001
Systolic blood pressure (mmHg%)	122.11 ± 14.90	123.21 ± 15.36	122.4 ± 15.04	124.08 ± 15.65	< 0.001
Diastolic blood pressure (mmHg%)	76.21 ± 10.02	76.17 ± 10.19	75.99 ± 10.26	76.37 ± 10.10	0.805
Fasting glucose (mg/dL%)	96.80 ± 23.36	97.67 ± 21.44	96.91 ± 21.37	98.49 ± 21.49	0.017
Total cholesterol (mg/dL%)	195.27 ± 41.10	194.21 ± 36.24	195.3 ± 35.50	193.04 ± 37	0.099
High-density lipoprotein (mg/dL%)	56.58 ± 32.52	56.61 ± 35.98	57.73 ± 40.36	55.39 ± 30.54	0.956
Chronic kidney disease (eGFR < 60 ml/min/1.73m2)	1,035,966 (10.87%)	734 (17.75%)	337 (15.74%)	397 (19.91%)	< 0.001

Continuous variables are expressed as means ± standard deviations. Regular physical activity is defined as having one or more sessions in a week with high (such as running, climbing, intense bicycle activities) or moderate physical activity (such as walking fast, tennis, or moderate bicycle activities).

*BMI* body mass index, *eGFR* estimated glomerular filtration rate, *PVC* premature ventricular contraction.

### New-onset AF

**Figure 2:**
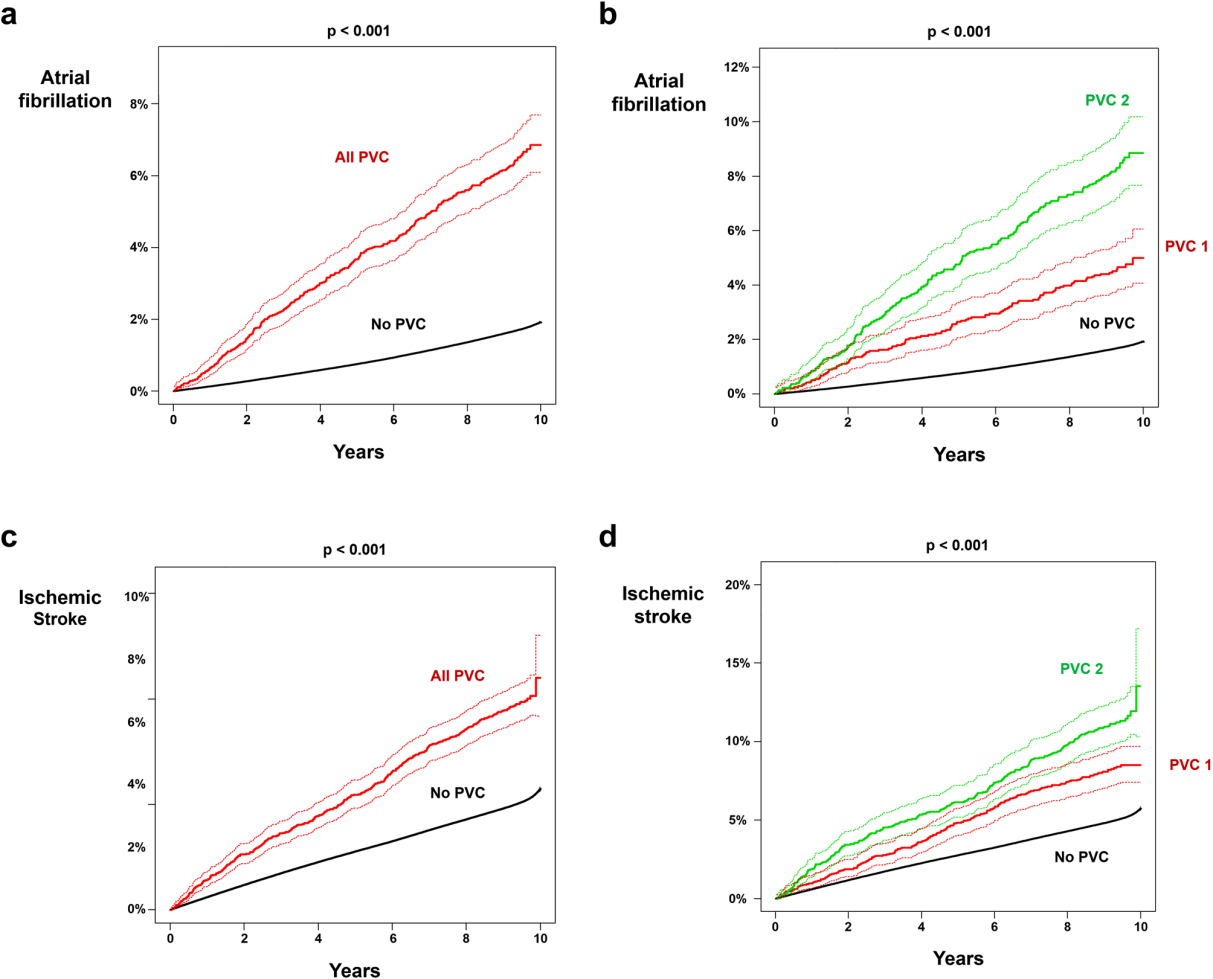
Impact of PVC on new-onset atrial fibrillation and ischemic stroke. Cumulative incidence curve analysis showed a significantly higher incidence of new-onset AF (**a**,**b**) and ischemic stroke (**c**,**d**) in people with PVC as compared with people without PVC. Dotted lines represent 95% confidence intervals. *PVC* premature ventricular contraction.

In people with no PVC, a total of 192,549 new-onset AF developed during the 87,256,944 person * year follow-up (incidence: 2.207). A total of 330 new-onset AF cases were observed in people with PVC during the 36,419 person * year follow-up (incidence: 9.061). The incidence of new-onset AF was even higher in people with PVC 2 with 205 new-onset AF cases during the 17,225 person * year follow up (incidence = 11.902) as compared with PVC 1 (incidence = 6.512; non-adjusted HR 1.848; 95% CI 1.480–2.308; p < 0.001). Kaplan–Meier curve analysis showed significantly higher incidence of new-onset AF in people with PVC as compared with people with no PVC (p < 0.001; Fig. 2a,b). Multivariate adjusted models also revealed a significantly increased risk of new-onset AF in people with PVC as compared with those without PVC (Table 2). Furthermore, people with PVC 2 had a significantly higher risk of new-onset AF as compared with PVC 1 (adjusted HR 1.493; 95% CI 1.195–1.865; p < 0.001). Subgroup analysis revealed a significant interaction between PVC and age (p < 0.001), with people < 65 years at greater risk of developing new-onset AF for having PVC (Supplementary Table S2).

**Table 2:** The risk of new-onset AF and ischemic stroke in people with PVC.

	n	Event number	Follow-up duration (person * year)	Incidence rate	Model 1	Model 2	Model 3	Model 4	Model 5
**Atrial fibrillation**									
No PVC	9,533,578	192,549	87,256,944	2.207	1 (Reference)	1 (Reference)	1 (Reference)	1 (Reference)	1 (Reference)
All PVC (PVC 1 + PVC 2)	4135	330	36,419	9.061	4.111 (3.690–4.580)	2.849 (2.557–3.173)	2.846 (2.555–3.171)	2.705 (2.428–3.013)	2.444 (2.180–2.741)
PVC 1	2141	125	19,194	6.512	2.956 (2.481–3.521)	2.178 (1.828–2.596)	2.195 (1.842–2.615)	2.149 (1.804–2.561)	1.917 (1.589–2.313)
PVC 2	1994	205	17,225	11.902	5.406 (4.714–6.199)	3.505 (3.057–4.020)	3.474 (3.029–3.984)	3.209 (2.799–3.680)	2.917 (2.525–3.369)
**Ischemic stroke**									
No PVC	9,533,578	451,246	86,019,868	5.246	1 (Reference)	1 (Reference)	1 (Reference)	1 (Reference)	1 (Reference)
All PVC (PVC 1 + PVC 2)	4135	374	36,219	10.326	1.966 (1.777–2.176)	1.23 (1.111–1.361)	1.235 (1.116–1.366)	1.160 (1.048–1.284)	1.166 (1.062–1.280)
PVC 1	2141	162	19,001	8.526	1.622 (1.391–1.892)	1.097 (0.941–1.280)	1.114 (0.955–1.300)	1.092 (0.936–1.274)	1.113 (0.967–1.281)
PVC 2	1994	212	17,219	12.312	2.355 (2.059–2.693)	1.356 (1.185–1.551)	1.345 (1.176–1.539)	1.218 (1.065–1.394)	1.211 (1.069–1.372)

Incidence rate is per 1000 person * years.

Model 1 is without multivariate adjustment.

Model 2 is adjusted for age and sex.

Model 3 is adjusted for age, sex, BMI, smoking status, alcohol consumption, and physical activity.

Model 4 is adjusted for age, sex, BMI, smoking status, alcohol consumption, physical activity, hypertension, diabetes, dyslipidemia, and chronic kidney disease.

Model 5 is adjusted for age, sex, BMI, smoking status, alcohol consumption, physical activity, hypertension, diabetes, dyslipidemia, chronic kidney disease, myocardial infarction, mitral stenosis, and aortic stenosis.

*AF* atrial fibrillation, *PVC* premature ventricular contraction.

### Ischemic stroke

In people with no PVC, 451,246 ischemic stroke events occurred during follow-up with an incidence of 5.246. A significantly higher incidence of ischemic stroke was observed in people with PVC (10.326). People with PVC 2 had significantly higher incidence of ischemic stroke as compared with people with PVC 1 (incidence = 12.312 vs. 8.526; non-adjusted HR 1.451; 95% CI 1.183–1.781; p < 0.001). Kaplan–Meier curve analysis showed a significantly higher incidence of ischemic stroke in people with PVC compared with people without PVC (p < 0.001; Fig. 2c,d). The increased risk of ischemic stroke in people with PVC was maintained after multivariate adjustment (Table 2). Those under 65 years and who were female were at greater risk of ischemic stroke for having PVC, suggesting a significant association with PVCs (Supplementary Table S3).

### New-onset AF and ischemic stroke

Among the 4135 people with PVC, 330 and 374 people developed new-onset AF and ischemic stroke, respectively (Table 3). New-onset AF developed in 72 people (19.3%) among the 374 people who had ischemic stroke during follow-up suggesting that an increased risk of new-onset AF may contribute to increased risk of ischemic stroke in people with PVC.

**Table 3:** New-onset AF and ischemic stroke.

	n	Stroke	Co-occurrence of AF and ischemic stroke
No PVC	9,533,578	451,246	45,983 (10.19%)
All PVC (PVC 1 + PVC 2)	4135	374	72 (19.25%)
PVC 1	2141	162	33 (20.37%)
PVC 2	1994	212	39 (18.40%)

*AF* atrial fibrillation, *PVC* premature ventricular contraction.

## Discussion

This study revealed an increased risk of new-onset AF and ischemic stroke in people with PVC. Various risk factors for AF have been reported in previous studies, but whether PVC could increase the risk of new-onset AF was unknown^17,18^. Our study is the first to report such an association. Due to sufficient sample size, we were able to perform various subgroup analyses and demonstrated that the risk of new-onset AF and ischemic stroke is more pronounced in people < 65 years. Since the identification of PVC was based on diagnostic codes, patients with PVC in our nationwide cohort are highly likely to be symptomatic patients seeking specific medical care for PVC. Therefore, our data can have practical implications for patients with PVC having outpatient or inpatient medical follow-ups. Heterogeneous duration of PVC originating from ICD-10 code based diagnosis can be problematic. To resolve this limitation, we excluded people with prior diagnosis of PVC during 2002 to 2008 and only those with newly diagnosed PVC in 2009 were included. Due to sufficient follow-up duration and large sample size, we were able to observe the influence of newly diagnosed PVC on AF and ischemic stroke.

### PVC and new-onset AF

Common risk factors exist between PVC and AF, e.g., age, hypertension, and diabetes mellitus. In our cohort, people with PVC were older and had higher prevalence of hypertension and diabetes, which are all risk factors associated with AF. However, increased risk of new-onset AF in people with PVC was statistically significant even after adjusting for these common risk factors. Conversely, people with PVC were less likely to be male in contrast with AF, which had male predominance. Body weight status, another important risk factor for AF, also did not differ between people with and without PVC. Our prior study showed no reliable association between dyslipidemia and AF, but the prevalence of dyslipidemia was higher in people with PVC than in those without PVC in this study.

People with PVC 2 had greater risk of new-onset AF compared with people with PVC 1. Diagnosis of PVC 2 was based on intensified criteria compared with PVC 1; therefore, PVC 2 might represent a more advanced form of PVC. Higher risk of new-onset AF in people with PVC 2 also support the association between PVC and new-onset AF.

The underlying mechanism linking PVC and increased risk of new-onset AF is not clear. Retrograde ventriculo-atrial conduction can occur with PVC. These retrograde atrial activations can act like an atrial ectopies originating from pulmonary veins. Reduced left ventricular function and subsequent increase in left atrial pressure can also contribute to increased risk of new-onset AF in people with. We have previously reported the impact of hypertension and diabetes on new-onset AF is more profound in young people as compared to the old^20^. Similarly, pronounced impact of PVC on new-onset AF and ischemic stroke in young people is another important finding of our study. The underlying mechanism of such interaction is not clear but may involve different characteristics of PVC in young vs. old age. Common genetic predisposition of PVC and AF in young people can also be the cause of such interaction.

### Ischemic stroke

Prior studies reported an association between PVC and ischemic stroke^14,15^. However, the underlying pathophysiology linking PVC and ischemic stroke is not fully elucidated. Not only AF but also premature atrial contraction has previously been reported to increase the risk of ischemic stroke^21^. Retrograde ventriculo-atrial conduction by PVC can act like a premature atrial contraction, which may be the underlying cause of the increased risk of ischemic stroke. Increased risk of AF in people with PVC can also contribute to the occurrence of ischemic stroke. Among the 4135 people with PVC, 330, 374, and 72 people developed new-onset AF, ischemic stroke, or both, respectively, suggesting a possible causal relationship between new-onset AF and ischemic stroke. Another hypothesis is concealed cardiomyopathy. Although we excluded people with prior diagnosis of heart failure, people with subclinical cardiomyopathy would not have been excluded. Premature ventricular contraction can be the first symptom of underlying subclinical cardiomyopathy in those people. When subclinical cardiomyopathy becomes clinical heart failure it can increase the risk of new-onset AF. If the underlying cardiomyopathy also affects atrial myocardium, it can also increase the risk of new-onset AF and ischemic stroke^22^. Adjusting the confounder effect of subclinical cardiomyopathy will be an important issue in future trials.

Our study raises the hypothesis that PVC can increase the risk of new-onset AF and ischemic stroke. Although people with PVC were older and had higher prevalence of hypertension, diabetes mellitus, and dyslipidemia, an association between PVC and ischemic stroke was shown after adjusting multiple parameters. Furthermore, when PVC were diagnosed with more intensified criteria (PVC 2), the incidence of ischemic stroke was higher (Table 2). Whether suppression of PVC with drugs or radiofrequency catheter ablation can reduce the risk of new-onset AF and ischemic stroke should be tested in future trials.

### Limitations

This study has several limitations. First, we were not able to obtain the burden of PVC measured by Holter monitoring. Currently, the K-NHIS database does not have any data regarding PVC burden. However, based on the number of inpatient or outpatient records for PVC, we classified PVC into PVC 1 or PVC 2. Diagnosis of PVC 2 was based on more intensified criteria and risk of new-onset AF, and ischemic stroke was higher in PVC 2 as compared with PVC 1. It is possible that that advanced form of PVC (PVC 2) are associated with more adverse events. Second, this study was based on a nationwide administrative database, and the results might have missing data due to coding inaccuracies. It is possible that people with asymptomatic PVC were not included in the PVC group. Although identification of PVC with ICD-10 codes (I49.3) using nationwide medical database is a novel approach, it needs further validation. Differences in baseline demographics originating from the retrospective nature of the study is another limitation. Third, our database was based only on East Asian people, and caution is required when applying our results to other ethnic groups. Fourth, we were not able to distinguish between paroxysmal (trigger type) and non-paroxysmal (substrate type) AF, which might have different pathophysiology. If retrograde ventriculo-atrial activation is responsible for triggering AF in people with PVC, trigger type AF rather than substrate type AF will be the predominant form of AF observed in people with PVC.

## Conclusions

Premature ventricular contractions might increase the risk of new-onset AF and ischemic stroke. Whether suppression of PVC with drugs or radiofrequency catheter ablation can reduce the risk of new-onset AF and ischemic stroke should be tested in future trials.

## Supplementary Information

Supplementary Information

## Data Availability

The data underlying this article are available in the article and in its online supplementary material.

## Abbreviations

AF: Atrial fibrillation
BMI: Body mass index
HR: Hazard ratio
ICD: International classification of disease
K-NHIS: Korean National Health Insurance Service
PVC: Premature ventricular contraction
ROC: Receiver operating characteristic
