# Supplementary material for: Premature ventricular contraction is associated with increased risk of atrial fibrillation: a nationwide population-based study
Source: Sci Rep. 2021 Jan 15;11:1601. doi: 10.1038/s41598-021-81229-0 (PMC7810887; doi:10.1038/s41598-021-81229-0)
Supplement: Supplementary file 1 — Supplementary Information [file 41598_2021_81229_MOESM1_ESM.docx]

**Premature Ventricular Contraction is Associated with Increased Risk of Atrial Fibrillation: A Nationwide Population-based Study**

Yun Gi Kim,^1^ Kyung-Do Han,^2^ Jong-Il Choi,^1^* Yun Young Choi,^1^ Ha Young Choi,^1^ Jaemin Shim,^1^ and Young-Hoon Kim^1^

^1^Division of Cardiology, Korea University College of Medicine and Korea University Anam Hospital, Seoul, Republic of Korea

^2^Department of Statistics and Actuarial Science, Soongsil University, Seoul, Republic of Korea

*Address for correspondence

Jong-Il Choi, MD, PhD, MHSc

Division of Cardiology, Korea University College of Medicine and Korea University Anam Hospital, Seoul, Republic of Korea

73 Goryeodae-ro, Seongbuk-gu, Seoul 02841, Republic of Korea

Tel: 82-2-920-5445

Fax: 82-2-927-1478

E-mail: [jongilchoi@korea.ac.kr](mailto:jongilchoi@korea.ac.kr)

**Brief title:** PVC and AF

**Total word count:** 4,756

The first two authors contributed equally to this work.

**Supplementary material table of contents**

**Supplementary Table S1.** Diagnostic codes used in this study.

**Supplementary Table S2.** Interaction analysis for new-onset AF.

**Supplementary Table S3.** Interaction analysis for ischemic stroke.

**Supplementary Table S1.** Diagnostic codes.

|  | ICD-10 codes |
| --- | --- |
| **Premature ventricular contraction** | I49.3 |
| **Atrial fibrillation** | I48 |
| **Heart failure** | I50 |
| **Ventricular tachycardia** | I47.0, I47.2 |
| **Ventricular fibrillation** | I49 |
| **Type 2 diabetes** | E11 – E14 |
| **Hypertension** | I10 – I13, I15 |
| **Dyslipidemia** | I78 |
| **Chronic kidney disease** | Based on creatinine checked during national health check-up |
| **Stroke** | I63, I64 |
| **Myocardial infarction** | I21, I22 |
| **Mitral stenosis** | I05.0 |
| **Aortic stenosis** | I35.0 |

**Supplementary Table S2.** Interaction analysis for new-onset AF.

| **Subgroup** | **PVC** | **n** | **Event number** | **Follow-up duration** | **Incidence** | **Multivariate model** | **p for interaction** |
| --- | --- | --- | --- | --- | --- | --- | --- |
| Age < 65 | No PVC | 8,506,282 | 119,225 | 78,499,958 | 1.519 | 1 (Reference) | < 0.001 |
|  | PVC | 3,263 | 204 | 29,294 | 6.964 | 3.422 (2.984 – 3.925) |  |
| Age ≥ 65 | No PVC | 1,027,296 | 73,324 | 8,756,986 | 8.373 | 1 (Reference) |  |
|  | PVC | 872 | 126 | 7,124 | 17.686 | 2.033 (1.707 – 2.421) |  |
| Male | No PVC | 5,306,708 | 116,155 | 48,323,335 | 2.404 | 1 (Reference) | 0.964 |
|  | PVC | 1,966 | 184 | 16,931 | 10.868 | 2.731 (2.364 – 3.155) |  |
| Female | No PVC | 4,226,870 | 76,394 | 38,933,609 | 1.962 | 1 (Reference) |  |
|  | PVC | 2,169 | 146 | 19,488 | 7.492 | 2.668 (2.268 – 3.139) |  |
| DM | No PVC | 8,781,043 | 162,127 | 80,602,216 | 2.011 | 1 (Reference) | 0.224 |
|  | PVC | 3,711 | 286 | 32,836 | 8.710 | 2.796 (2.49 – 3.14) |  |
| Non-DM | No PVC | 752,535 | 30,422 | 6,654,728 | 4.572 | 1 (Reference) |  |
|  | PVC | 424 | 44 | 3,582 | 12.282 | 2.224 (1.654 – 2.989) |  |
| HTN | No PVC | 7,207,504 | 95,411 | 66,446,015 | 1.436 | 1 (Reference) | 0.138 |
|  | PVC | 2,189 | 107 | 19,764 | 5.414 | 2.889 (2.39 – 3.492) |  |
| Non-HTN | No PVC | 2,326,074 | 97,138 | 20,810,929 | 4.668 | 1 (Reference) |  |
|  | PVC | 1,946 | 223 | 16,655 | 13.389 | 2.62 (2.298 – 2.988) |  |
| CKD | No PVC | 8,497,612 | 142,195 | 78,204,825 | 1.818 | 1 (Reference) | 0.434 |
|  | PVC | 3,401 | 226 | 30,358 | 7.445 | 2.797 (2.455 – 3.186) |  |
| Non CKD | No PVC | 1,035,966 | 50,354 | 9,052,119 | 5.563 | 1 (Reference) |  |
|  | PVC | 734 | 104 | 6,061 | 17.160 | 2.529 (2.086 – 3.065) |  |

AF: atrial fibrillation; CKD: chronic kidney disease; DM: diabetes mellitus; HTN: hypertension; PVC: premature ventricular contraction.

**Supplementary Table S3.** Interaction analysis for ischemic stroke.

| **Subgroup** | **PVC** | **n** | **Event number** | **Follow-up duration** | **Incidence** | **Multivariate model** | **p for interaction** |
| --- | --- | --- | --- | --- | --- | --- | --- |
| Age < 65 | No PVC | 8,506,282 | 248,881 | 77,879,742 | 3.196 | 1 (Reference) | 0.002 |
|  | PVC | 3,263 | 196 | 29,385 | 6.670 | 1.36 (1.182 – 1.565) |  |
| Age ≥ 65 | No PVC | 1,027,296 | 202,365 | 8,140,126 | 24.860 | 1 (Reference) |  |
|  | PVC | 872 | 178 | 6,834 | 26.046 | 1 (0.863 – 1.158) |  |
| Male | No PVC | 5,306,708 | 232,716 | 47,781,006 | 4.871 | 1 (Reference) | 0.041 |
|  | PVC | 1,966 | 159 | 17,148 | 9.272 | 0.999 (0.856 – 1.168) |  |
| Female | No PVC | 4,226,870 | 218,530 | 38,238,863 | 5.715 | 1 (Reference) |  |
|  | PVC | 2,169 | 215 | 19,071 | 11.274 | 1.304 (1.141 – 1.491) |  |
| DM | No PVC | 8,781,043 | 359,246 | 79,656,334 | 4.510 | 1 (Reference) | 0.133 |
|  | PVC | 3,711 | 310 | 32,750 | 9.466 | 1.199 (1.073 – 1.34) |  |
| Non-DM | No PVC | 752,535 | 92,000 | 6,363,535 | 14.457 | 1 (Reference) |  |
|  | PVC | 424 | 64 | 3,469 | 18.448 | 0.995 (0.778 – 1.271) |  |
| HTN | No PVC | 7,207,504 | 203,307 | 65,953,751 | 3.083 | 1 (Reference) | 0.243 |
|  | PVC | 2,189 | 114 | 19,697 | 5.788 | 1.249 (1.039 – 1.5) |  |
| Non-HTN | No PVC | 2,326,074 | 247,939 | 20,066,118 | 12.356 | 1 (Reference) |  |
|  | PVC | 1,946 | 260 | 16,522 | 15.737 | 1.126 (0.997 – 1.272) |  |
| CKD | No PVC | 8,497,612 | 305,975 | 77,414,535 | 3.952 | 1 (Reference) | 0.408 |
|  | PVC | 3,401 | 238 | 30,331 | 7.847 | 1.204 (1.06 – 1.367) |  |
| Non CKD | No PVC | 1,035,966 | 145,271 | 8,605,333 | 16.882 | 1 (Reference) |  |
|  | PVC | 734 | 136 | 5,889 | 23.095 | 1.09 (0.921 – 1.289) |  |

AF: atrial fibrillation; CKD: chronic kidney disease; DM: diabetes mellitus; HTN: hypertension; PVC: premature ventricular contraction.
